# Cannibalism and activity rate in larval damselflies increase along a latitudinal gradient as a consequence of time constraints

**DOI:** 10.1186/s12862-017-1010-3

**Published:** 2017-07-14

**Authors:** Szymon Sniegula, Maria J. Golab, Frank Johansson

**Affiliations:** 1 Department of Ecosystem Conservation, Institute of Nature Conservation, Polish Academy of Sciences, al. Mickiewicza 33, 31-120, Krakow, Poland; 2 Department of Ecology and Genetics, Uppsala University, SE-751 05 Uppsala, Sweden

**Keywords:** Abiotic constraints, Feeding, Phenotypic plasticity, Photoperiod, Predation, Temperature, Latitude

## Abstract

**Background:**

Predation is ubiquitous in nature. One form of predation is cannibalism, which is affected by many factors such as size structure and resource density. However, cannibalism may also be influenced by abiotic factors such as seasonal time constraints. Since time constraints are greater at high latitudes, cannibalism could be stronger at such latitudes, but we know next to nothing about latitudinal variation in cannibalism. In this study, we examined cannibalism and activity in larvae of the damselfly *Lestes sponsa* along a latitudinal gradient across Europe. We did this by raising larvae from the egg stage at different temperatures and photoperiods corresponding to different latitudes.

**Results:**

We found that the more seasonally time-constrained populations in northern latitudes and individuals subjected to greater seasonal time constraints exhibited a higher level of cannibalism. We also found that activity was higher at north latitude conditions, and thus correlated with cannibalism, suggesting that this behaviour mediates higher levels of cannibalism in time-constrained animals.

**Conclusions:**

Our results go counter to the classical latitude-predation pattern which predicts higher predation at lower latitudes, since we found that predation was stronger at higher latitudes. The differences in cannibalism might have implications for population dynamics along the latitudinal gradients, but further experiments are needed to explore this.

**Electronic supplementary material:**

The online version of this article (doi:10.1186/s12862-017-1010-3) contains supplementary material, which is available to authorized users.

## Background

Predation is ubiquitous, and theory as well as empirical studies suggests that predation is higher at lower latitudes [1–3]. However, we have little knowledge on how intraspecific predation, more commonly referred to as cannibalism, varies along a latitudinal gradient, but see Pereira et al. [4]. Cannibalism may be density dependent (such that a higher risk is achieved with more potential cannibals), or density independent where cannibalism varies with other environmental variables besides density of conspecifics [5, 6]. In this is study we focus on the density independent cannibalism driven by seasonal time constraints along a latitudinal gradient.

Cannibalism, defined as the killing and eating of individuals of the same species, is common in many organisms [7]. There are several potential advantages. First, there could be a direct, greater nutrient effect from consumption, and hence increased growth and development [8]. Second, there could be an indirect advantage in that a conspecific competitor is eliminated, thus reducing competition in the future; growth and development are also enhanced thereby [9, 10]. Cannibalism does, however, also have certain disadvantages. For example, there is a risk that the potential cannibal becomes the victim [9, 11]. Moreover, there is a risk of cannibalising a related individual, which will decrease inclusive fitness [12]. Finally, there is also a higher risk of pathogen and parasite infection when cannibalising a conspecific compared to preying on a non-specific [13]. Hence, we expect cannibalism to vary with environmental factors and to be more common when the benefits are greater than the costs.

Usually, cannibalism is more common when individuals differ in size, but cannibalism among similarly sized individuals also occurs [7, 14, 15]. In general, cannibalism is higher at high population densities because encounters between individuals increase [16]. Also, resource levels could affect cannibalism intensity. As food levels go down, body condition also deteriorates, which affects cannibalism [17]. For example, when prey is scarce, starvation levels increase, which could increase cannibalism [18]. The higher level of cannibalism in starved individuals is believed to be a result of higher levels of aggression [18]. However, it could also be a result of more encounters between individuals since the encounter rate should go up when starved animals are actively searching for food [19, 20].

One factor that could affect cannibalism is the time constraints that are imposed by seasonality (but see [10, 21]). Seasonal time constraints are set by the variation in temperature that cause the annual cycles of winter and summer [22, 23], and in most situations day length is an environmental cues that can be used for estimation length of the potential season. Note that at higher latitudes a long day length the weeks before and after summer solstice, although it allows for more foraging and growth (since more hours of light are available), actually is a cue that signals seasonal time constraints. Such constraints are common in organisms with complex life cycles, such as insects and amphibians [24–26]. Seasonal time constraints usually increase with latitude and altitude because the time available for growth and development decrease with these variables [27]. Similarly, time constraints are stronger in organisms with obligate one-year life cycles, since they have to complete their life cycle within one growing season. An organism with a flexible life cycle could add another year of growth and development if the season is too short, thereby lessening the time constraint. Since cannibalism may increase growth and development by the addition of extra nutrients and the reduction of potential competitors [9, 10], we can hypothesise that cannibalism should increase with the intensity of time constraints. For example, De Block and Stoks [10] found that time constrained damselfly larvae increased cannibalism, which resulted in faster development rate and growth. Hence, along a latitudinal gradient in a species with an obligate one-year life cycle, cannibalism should be stronger in the season time constrained north than in the south. Indeed, many studies have shown that northern populations do have a higher growth and faster development than southern populations, e.g. [27–30], but no studies have examined how cannibalism varies along a latitudinal gradient.

In this study we focused on non-filial and non-sexual cannibalism in larvae of the damselfly *Lestes sponsa* (Hansemann). The main aim of the study was to examine how cannibalism changes in damselfly larvae along a latitudinal gradient. We compared populations from three latitudes: south, centre and north. We predict a higher level of cannibalism in the northern populations and a higher level of cannibalism under more time-constrained environmental conditions. Since cannibalism increases with activity we also assessed this behaviour in the larvae. We predict a higher level of activity where time constraints are greater, since these larvae have to search more actively for food. We also predict a positive correlation between cannibalism and activity.

## Methods

The damselfly *Lestes sponsa* (Hansemann) has a one-year obligate life cycle consisting of a long-lived aquatic larval stage (2–3 months), during which the main part of growth and development occurs, and a relatively short-lived terrestrial adult stage (days to weeks), when reproduction takes place. The European distribution of *L. sponsa* ranges from southern France to northern Sweden [31].

We examined cannibalism in *L. sponsa* under two environmental conditions: a common garden experiment and a simulated natural condition experiment. For both experiments eggs from two replicate lake populations per latitude were collected using a common method [32]. By sampling and analysing two populations per latitude (random population effect) we decreased the influence of lake specific environmental effects. The populations were situated in southern France (43°N), Poland (54°N) and northern Sweden (66°N). Estimates of population densities at these populations are given in Sniegula et al. [30], and in general the densities are lower in the north. The distances between the replicate populations ranged from 5 to 151 km and they were all permanent waters. The temperatures that the eggs and larvae experienced during both experiments were derived from the FLake lake model [33]. For a detailed description of the FLake model, see Sniegula et al. [30]. All applicable institutional and/or national guidelines for the care and use of animals were followed.

### Cannibalism: Common garden experiments

For the common garden experiment, eggs were collected on 28 June 2013 at the southern latitude, 28 July 2013 at the central latitude, and 3 Aug 2013 at the northern latitude. We sampled 25, 25 and 50 egg clutches from the southern, central and northern latitudes, respectively. These egg clutches were sampled by catching mating females and putting them separately in small glass jars with a moist filter paper wherein females deposited their eggs. Upon arrival at the laboratory, we placed the eggs in separate plastic containers (12 × 8 cm, height 5 cm) filled with 250 ml of dechlorinated tap water and put them in a climate chamber with temperature 21.9 °C and photoperiod L-D 19:25–4:35. We set this temperature because individuals from each latitude experience it for at least several hours a day during the main growing season in nature [30]. The photoperiod reflected the longest day length at the mid-latitude across our sampled latitudes, 55°N.

After 17 days we initiated winter conditions. We lowered the temperature to 15 °C while maintaining the existing photoperiod regime. The following day we further reduced the temperature to 5 °C and set the light level to 24 h of darkness to simulate winter conditions, which were simulated for 28 days. Thereafter the eggs were transferred to a climate chamber with a constant temperature of 21.9 °C and L-D 19:25–4:35. Importantly, the L-D 19:25–4:35 triggered the eggs from the central and southern latitudes to hatch very synchronously and the northern latitude eggs to hatch synchronously enough for there to be a sufficient number of individuals for the cannibalism experiment [30]. Note that due to different sampling dates (see above), the cannibalism experiment started at different days for each latitude.

The northern latitude eggs hatched 14 days (2 Oct 2013), the central latitude eggs 7 days (26 Sept 2013) and the southern latitude eggs 5 days (27 Aug 2013) after we had transferred them to the climate chamber with the constant summer temperature and light regime. Immediately after hatching, the eggs from all the clutches originating from the two populations per latitude were mixed in a plastic bucket (5 l volume). Thereafter, we randomly divided the hatchlings into groups of ten individuals and introduced them into plastic containers (12 × 8 cm, height 5 cm) filled with 400 ml of dechlorinated tap water. The number of replicates was 50, 25 and 25 for northern, central and southern latitudes, respectively. Each container had two strips of nylon net (14 cm long and 3 cm wide) crossing each other in the centre of the container. The larvae were fed twice a day for 14 days with *Artemia* nauplii (the mean *Artemia* portion: 113.25, SD: 10.25, *N* = 10). We did not observe cannibalism during this time. After 14 days, we reduced the feeding rate to three times a week: Monday, Wednesday and Friday. To check for cannibalism, we counted the individuals every fourth day, and for the statistical comparisons of cannibalism we used the numbers of individuals cannibalized on days 12 and 28, corresponding to an age of 26 and 42 days, respectively. We estimated the rate of cannibalism by taking into account missing and dead individuals with signs of attack (extrinsic causes of death). Damselfly and dragonfly larvae are not scavengers, and only strike against moving prey [34]. We did not observe individuals that died due to intrinsic effects, e.g. due to developmental errors.

### Cannibalism: Simulated natural conditions

For the simulated natural condition experiment we collected eggs on 15 June 2015 in southern populations, on 5 Aug 2015 in central populations and on 12 Aug 2015 in northern populations. The populations and the egg sampling method were the same as in the common garden experiment. Similarly, the cannibalism experiment on different latitudes started at different days due to different sampling dates across latitudes. We sampled 20 clutches from the two replicate populations per latitude. After arrival at the laboratory, the eggs were placed in plastic containers filled with 250 ml of dechlorinated tap water and put in climate chambers with temperatures and photoperiods simulating late summer conditions at each latitude of origin of the populations, i.e. the environmental conditions of the source animals. For the northern, central and southern latitudes the temperatures were 19.2 °C, 21 °C and 24.8 °C, respectively, and the photoperiods were L-D 20:57–3:03, 17:38–6:22, and 16:31–7:29, respectively.

Seventeen days after the eggs had been placed in the climate chambers, we initiated simulated winter conditions. We lowered the temperature to 15 °C while maintaining the existing photoperiod regime. The following day (29 Aug 2015, 22 Aug 2015 and 2 July 2015 for the northern, central and southern latitudes, respectively) we reduced the temperature to 5 °C and set the photoperiod to 24 h of darkness. Winter conditions were simulated for 48, 50 and 50 days for northern, central and southern latitudes, respectively.

Immediately after the end of the simulation of winter conditions, as in the common garden experiment, all the eggs were transferred to another climate chamber with temperature 21.9 °C and photoperiod L-D 19:25–4:35. We knew from the previous experiment that such a temperature and photoperiod would trigger synchronous hatching [26]. The northern eggs hatched 10 days (27 Oct 2015), the central eggs 4 days (16 Oct 2015) and the southern eggs 2 days (24 Aug 2015) after the simulation of winter conditions had ended. Directly after hatching, 10 hatchlings were chosen at random from each container (= egg clutch) and transferred to another container (12 × 8 cm, 5 cm high) with 400 ml of dechlorinated tap water with two nylon net strips crossing the inner part of the container. The number of replicates was 20, 20 and 20 for northern, central and southern latitudes, respectively. On the same day, these hatchlings were moved back to their original chambers with programmed spring conditions.

The simulation of spring conditions reflected the dates when the temperature exceeded 12 °C at each latitude: northern latitude, 30 May, 14 °C; central latitude, 25 Apr, 13.3 °C; southern latitude, 4 Apr, 13.8 °C. To simulate the progression of spring and summer, we simulated weekly changes of temperature and photoperiod until the end of the experiment (Additional file 1: Fig. A1). The larvae were fed twice a day with *Artemia* nauplii (mean nauplii portion: 121.83, SD: 9.48, *N* = 10). As in the common garden experiment, we reduced the feeding rate to three times per week after 14 days. To check for cannibalism, individuals were counted every fourth day, but for the statistical comparisons of cannibalism we used the number of individuals cannibalized on days 12 and 28, corresponding to an age of 26 and 42 days, respectively. We estimated the rate of cannibalism by taking into account missing and dead individuals with extrinsic causes of death. No individuals died as a result of intrinsic causes. In this experiment we did not mix individuals originating from different families, which allowed us to associate activity level and cannibalism rate at the family level. Hence, all observed cannibalism was within a family (clutch).

### Experiment: activity

Activity was estimated on the same group of larvae, i.e. originating from the same egg clutches as were used for the cannibalism experiment performed in the simulated natural conditions in 2015. On the day when the eggs hatched in the climate chamber at 21.9 °C and photoperiod L-D 19:25–4:35 (see above), we randomly chose 8 individuals from each family and placed them individually in round plastic containers (diameter 7 cm, height 4 cm) filled with dechlorinated tap water. Thereafter we reared the larvae under two conditions: 1) common garden conditions (larvae reared in the same chamber as where they had hatched), and, 2) simulating the native latitudinal temperature and photoperiod conditions as described above. We kept individual larvae in separate chambers until the larval activity experiment started, which took an average of 69.4, 70.8 and 58.8 days, respectively, for southern, central and northern latitude populations grown in common garden conditions, and 85.2, 77.0 and 62.5 days for southern, central and northern latitude populations grown in simulated conditions. Larvae were fed every morning and afternoon with *Artemia* nauplii (mean nauplii portion: 121.83, SD: 9.48, *N* = 10). Three days after the larvae had moulted into the final instar, we ran the behaviour observations. The larvae were not fed the day prior to the activity observations. On the first day of the activity observation, each larva was removed from the climate chamber and individually placed in a bigger container, 12 × 8 cm, 5 cm high, with a grid of 2 × 2 cm drawn on the bottom. Larvae were allowed to acclimate in the container 30 min before the observations started. After these 30 min 10 laboratory cultured *Daphnia* sp. of standardized size were introduced and the behavioural observation started. Depending on the number of larvae that reached the appropriate stage on a given day, we observed between 2 and 10 larvae per day. Larval moves were monitored continuously for 20 min by the same person. Activity was scored by measuring movement in relation to the grid pattern (2 × 2 cm squares) at the bottom of each container. A move was recorded when a larva had moved its head (Additional file 1: Figure A2). From these observations we calculated the total number of moves, the total distance moved, and the mean distance per move. All the experiments were run between 11:30 and 15:00. We did not measure activity on the larvae from the cannibalism experiment, since the larvae that were left at the end of the cannibalism experiment differed in age and hence the number of moults they had gone through. Nevertheless, behavioural studies on activity of damselfly larvae show that larvae differ in their personality and that such larval differences are consistent over time, all else being equal. For example, Brodin [35] showed that *Lestes congener* individuals remained active over time compared with their conspecifics, independent of state or predator treatment. Similarly, general activity did not change among treatments in *Lestes sponsa* larvae over a four week period in an outdoor field experiment [36]. We therefore suggest that the difference in the experimental design between the cannibalism and the activity estimates did not bias our results.

### Statistical methods

For all the statistical analyses we used R 3.3.2 [37]. For all models we used Wald *χ* ^2^ statistics to test for fixed effects (car package, [38]) and then, if needed, assessed significance of the differences among levels by changing reference level of the categorical variable (latitude). This method does not include multiple testing, since all variables are already included in the model (thus, differences are corrected for all other factors) [39]. In the common-garden experiment we used generalized linear model GLM (glm function, [39]) with binomial error distribution with latitude (three levels) and time in days (two levels), and the interaction between the two as explanatory variables to analyse the difference in number of larvae cannibalized across experimental groups over time. For the simulated natural conditions experiment we used the same model structure to analyse the difference in number of larvae cannibalized over time, except that we added population within region as random effect and run generalized linear mixed model with binomial error distribution, GLMM (glmer function from lme4 package, [40]).

We used GLM function with quasi-Poisson error distribution to account for the overdispersion to analyse the differences in the number of days until one larva was left in each container. Time (days) was a response and latitude was an explanatory variable. We did not include rearing treatment (simulated natural and common-garden conditions) as a factor since the food level and the way we mixed individuals between families differed between these treatments. Hence, we ran separate tests for common garden and simulated conditions.

To analyse the number of moves we used GLMM model (glmer function [40]). To analyse the mean distance per move and the total distance moved we used linear mixed models (lmer function [38]). We log-transformed the variables mean distance per move and the total distance moved to improve the normality of errors. Latitude was a factor and population within a region was a random effect for these three response variables. We used the same model structures when analysing data for simulated and common garden conditions.

To explore whether there was a relationship between cannibalism and activity we used GLM, and to check whether this relationship differed across latitudes we used GLMM (number of moves) and linear mixed model function (mean and total distance moved) (glmer and lmer functions, respectively) [40]. Cannibalism rates were response variables, activity traits were covariates and latitude was a factor. Population within region was a random effect. These relationships were estimated with larvae from the simulated natural condition experiment. We used mean family values, since activity is difficult to estimate during the cannibalism experiments. Hence, we assumed that activity and the cannibalism rate are associated within sibs, and used the average family value on activity and cannibalism as our replicated unit.

## Results

### Cannibalism: Simulated natural conditions

**Fig. 1:**
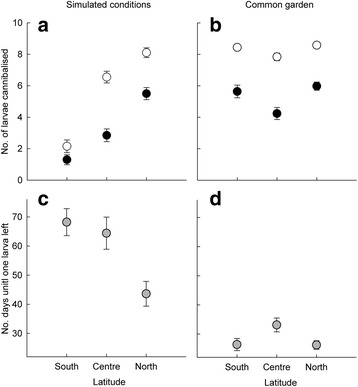
**a**, **b** Number of Lestes sponsa larvae cannibalized at day 12 (*filled symbols*) and day 28 (*open symbols*) and **c**, **d** number ofdays until only one larva was left in the experiments under simulated and common garden environmental conditions. *Error bars* are ±SE

The number of larvae cannibalized increased with latitude, and over time (Fig. 1a), and the probabilities of survival of larvae from northern, central and southern populations until day 12 were 0.45, 0.72 and 0.87, respectively, and until day 28 were 0.19, 0.34 and 0.78, respectively. These patterns were significant since the GLMM showed a significant latitude effect (*χ* ^2^_2_ = 198.259, *P* < 0.001) and also a significant change over time (*χ* ^2^_1_ = 79.197, *P* < 0.001). The interaction between latitude and time was significant (*χ* ^2^_2_ = 7.609, *P* = 0.022), indicating higher increase in cannibalism intensity over time in northern populations. In addition, there was a significant difference in the number of larvae cannibalized between all three latitudes (all *P*-values <0.05).

The number of days until one larva was left decreased with latitude and thus showed the same latitudinal pattern as for the number of larvae cannibalized (Fig. 1c). Hence, the cannibalism rate at the northern latitude was higher compared to the south (GLM: *χ* ^2^_2_ = 125.31, *P <* 0.001). The GLM showed that northern latitude differed significantly from the central (*P* = 0.002) and southern (*P* < 0.001) ones, but that the central and southern latitudes did not differ from one another (*P* = 0.604).

### Cannibalism: Common garden

There was a significant latitudinal effect (GLM: *χ* ^2^_2_ = 26.669, *P <* 0.001; Fig 1b) and temporal effect (GLM: *χ* ^2^_1_ = 205.632, *P* < 0.001) on the number of larvae cannibalized, but no interaction effect (GLM: *χ* ^2^_2_ = 0.616, *P =* 0.735). Curiously, cannibalism was higher in northern and southern populations than in central latitudes (both *P* < 0.001), but cannibalism in northern and southern populations did not differ from one another (*P* = 0.305). The probabilities of survival of larvae from northern, central and southern populations until day 12 were 0.40, 0.58 and 0.44, respectively, and until day 28 they were 0.14, 0.22 and 0.16, respectively.

There was an overall significant effect on the number of days until one larva remained (GLM: *χ* ^2^_2_ = 30.078, DF = 2, *P* < 0.001; Fig. 1d). There was a significant difference between northern and central, and between central and southern latitudes (*P* < 0.036, for both comparisons), but no difference between northern and southern latitudes (*P* = 0.951).

### Activity

**Fig. 2:**
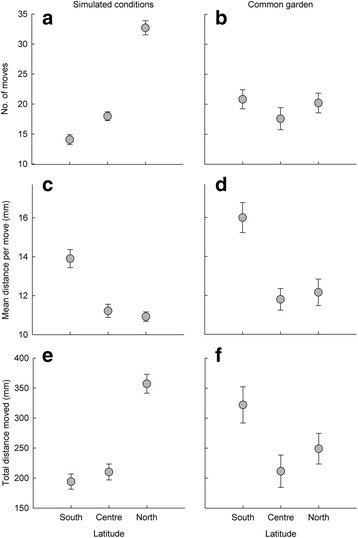
**a**, **b** Number of moves, **c**, **d** mean distance per move, and **e**, **f** total distance moved (±SE) in the experiment under (**a**, **c**, **e**) simulated natural light and temperature conditions and (**b**, **d**, **f**) in a common garden experiment with a light and temperature environment simulating a constant average over latitude and time

In the simulated conditions experiment the number of moves differed between latitudes (χ^2^_2_ = 413.2, *P <* 0.001). Number of moves was high in the northern latitude, low in the southern latitude and intermediate in the central latitude population (Fig. 2a). There was significant difference between latitudes (*P* < 0.001 of all pairwise comparisons). In the common garden experiment there was no difference in the number of moves between the populations from the different latitudes (*χ* ^2^_2_ = 2.056, *P* = 0.358). Neither of the latitudes differed between each other (all *P*-values ≥0.172) (Fig. 2b).

The mean distance per move was high in southern populations under southern conditions and low in northern populations under northern conditions, and intermediate for the central populations (Fig. 2c). There was overall significance of these differences (χ^2^_2_ = 11.863, *P* = 0.003). There was a borderline difference between northern and southern latitudes, and between central and southern latitudes (*P* = 0.055 and *P* = 0.065, respectively) and no difference between northern and central latitudes (*P* = 0.858). A similar pattern with respect to latitude was found in the common garden experiment (*χ* ^2^_2_ = 22.844, *P* < 0.001; Fig. 2d). There was a difference between northern and southern latitudes (*P* < 0.001), and between central and southern latitudes (*P* < 0.001) and no difference between northern and central latitudes (*P* = 0.708). The total distance moved was high in northern populations under northern conditions, low in southern populations under southern conditions and intermediate in central populations under the central latitude conditions (Fig. 2e), and, in general, these differences between populations were statistically significant (*χ* ^2^_2_ = 31.812, *P* < 0.001). Northern latitude differed from central and southern latitude (*P* = 0.021 and *P* = 0.013, respectively), but there was no statistical difference between central and southern latitude (*P* = 0.509). Overall, in the common garden experiment there was a significant difference in the total distance moved between populations (χ^2^_2_ = 8.579, *P* = 0.014; Fig. 2f). However, when we compared parameter estimates for each level of the variable latitude, we found that there was no difference between northern and southern latitude (*P* = 0.170), no difference between northern and central latitude (*P* = 0.318), and borderline difference between central and southern latitudes (*P* = 0.053).

### Correlation between cannibalism and activity

**Fig. 3:**
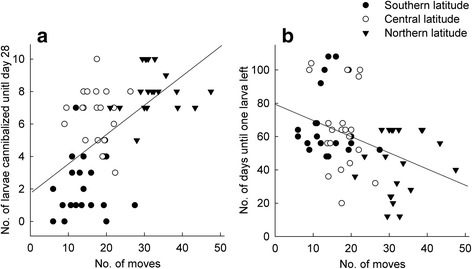
**a** The number of larvae cannibalized by day 28 and **b** the number of days until one larva was left, regressed against the number of larval moves during the experiment simulating natural conditions. The regression lines are based on the studied populations at all latitudes: adjusted *R* ^2^ = 0.327 and *P* < 0.001, and adjusted *R* ^2^ = 0.142 and *P* = 0.002 for the (**a**) and (**b**) figures, respectively

There was a significant effect of the number of moves on the number of larvae cannibalized when latitude was not included in the model (GLM: χ^2^_*1*_ = 30.477, *P* < 0.001; Fig 3a). When the significant effect of latitude (χ^2^_2_ = 40.555, *P* < 0.001; Fig 3a) was included in the model, the number of moves became non-significant (GLMM: χ^2^_*1*_ = 0.0001, *P* = 0.993). The number of days until one larva was left was significantly affected by the number of moves (GLM: χ^2^_1_ = 92.82, *P <* 0.001; Fig 3b), but when the significant effect of latitude (χ^2^_2_ = 15.997, *P* < 0.001) was included in the model, the number of moves turned to no significant (χ^2^_1_ = 0.601, *P* = 0.438). These results suggest that the correlations between cannibalism and activity were not statistically detectable at the population level, but instead were detectable across populations, i.e. at the species level. We also used models to examine the relationship between the mean distance moved and the number of larvae cannibalized, and the total distance moved and the number of larvae cannibalized (in both cases using data on the number of larvae cannibalized until day 28 and until one larva was left), and to examine whether latitudes affected cannibalism. The results from these models showed the same patterns as when using the mean number of moves as a response variable. The numerical results and figures are given in Additional file 1: Table A1, Figure A3.

## Discussion

We found support for our predictions that cannibalism should increase with stronger time constraints, because cannibalism was higher at northern latitude conditions. When northern populations were raised at northern latitude light and temperature conditions, cannibalism was significantly more intense than in the southern populations raised under southern latitude light and temperature conditions. This experiment with simulated environmental conditions does not tell us whether the observed higher level of cannibalism in the north is a fixed genetic effect for more cannibalism or an induced effect caused by the light conditions which simulates a shorter season. However, our common garden experiment suggests that the observed difference is not a pure genetic effect because cannibalism patterns was not similar to the simulated conditions experiment. The common garden experiment used a light regime that simulated a late season photoperiod for newly hatched larvae. Thus, this photoperiod, i.e. the day length, was an environmental cue for seasonal time constraints. This photoperiod simulated a perceived time constraint effect, being high for the southern, intermediate for the central and low (but still time-constrained) for the northern populations (northern populations would have needed a much longer daylight period to simulate very strong time constraints). If it was driven by phenotypic plasticity the southern damselflies would have had the highest level of cannibalism, the central the intermediate and the northern the lowest, but we did not observe this. Nevertheless, our results clearly show that *L. sponsa* larva reared at environmental conditions simulating natural temperature and light regimes at northern latitudes have higher cannibalism, compared to larvae raised at temperature and light conditions simulating those at southern latitude. However, more experiments are needed to evaluate how much of this is due to genetic and phenotypic plasticity, since northern population did not differ from southern ones in the common garden experiment.

The stronger cannibalism probably accelerates development and growth, although we have no direct evidence for this. However, in a manipulation experiment, De Block and Stoks [10, 21] found that a time-stressed population of *Lestes viridis* increased cannibalism, and that this cannibalism was correlated with a faster development rate and growth, as a consequence of greater food intake and less intraspecific competition. Such effects are probably adaptive in organisms at high latitudes, and can lead to a larger size at emergence [19, 36].

Cannibalism was mirrored in the pattern of the number of moves and the total distance moved, such that a high activity was correlated with a high level of cannibalism. However, activity and cannibalism were not associated within latitude. Nevertheless, the correlation across latitudes suggests that part of the cannibalism rate is a consequence of more frequent encounters (see also [20]). Larvae that are more time-constrained probably search more actively for prey, which in turn results in a higher encounter rate with conspecifics, and this potentially results in cannibalism. In support of this, Johansson [19] found that activity increased with lower prey density and that this resulted in a higher level of cannibalism in several species of odonate larvae. However, a higher level of cannibalism in more time-constrained individuals could also be an effect of a more general aggressive behaviour per se. Since we only had one alternative prey available in our experiment we cannot rule out the possibility that a general predation effect intensifies faster than cannibalism. However, one alternative prey (*Artemia*) was present during the experiment and despite this we observed higher cannibalism in northern environmental conditions. An interesting extension of our experiment would have been to provide another damselfly species as an alternative prey.

Nevertheless, the absence of a significant effect of activity on cannibalism when latitude was included in the model, suggests that the relationship between cannibalism and activity was not detectable within latitudes. This implies, that other factors besides activity also might contribute to differences in cannibalism along the latitudinal gradient. For example, more time-constrained animals might be more aggressive because they need more energy to develop and grow faster. Such energy effects of cannibalism was found in spiders where cannibalism was higher in starved larvae compared to non-starved ones [18].

In contrast to the number of moves and distance moved, we found that the mean distance moved was not positively correlated with cannibalism. In fact, it went in the opposite direction for the northern population under simulated conditions, suggesting that it is not important for cannibalism intensity. Northern individuals moved many times and at very short intervals, suggesting a very intensive hunt for prey. This very intensive movement over very short distances could lead to a high encounter rate between potential cannibals if during their first encounters they perceive each other as prey. However, movements and distance moved and their relationship with potential prey is dependent on the density of potential prey and activity of the prey and hence more work is needed to understand how cannibalism is affected by activity and density at a mechanistic level.

Interestingly, we found that the level of cannibalism and activity in southern populations raised in the common garden conditions were as a high as in the northern populations. We interpret this result as an effect of a very strong time constraint on these southern populations under these environmental conditions. The simulated photoperiod mimics a very late season for the southern population, and hence the larvae experience a strong time constraint, which should speed up their development, activity and cannibalism. In contrast, for the northern populations the common garden photoperiod, though simulating a time constraint, through a day length environmental cue, was much less strong. Had we used a longer photoperiod, simulating a stronger time constraint for the northern populations, these populations may have displayed a higher level of cannibalism and activity compared to the southern populations. We also found that number of days until one larvae was left was much shorter in the common garden experiments. This was probably an effect of temperature since temperature was high from the beginning of the experiment in the common garden conditions. Predation rate is temperature dependent in many invertebrates and peaks at optimal temperature [41]. Our comparison was only across latitude and not between the common garden and simulated conditions and therefore this temperature effect should have little impact on the interpretation of our result.

## Conclusions

In this study we showed that more time-constrained populations in northern latitudes and individuals subjected to higher time constraints exhibited a higher level of cannibalism. Such higher levels of cannibalism in time-constrained animals have been observed in other studies. For example, studies that manipulated the perceived time available for growth within a season found higher levels of cannibalism in time-constrained damselfly larvae [10, 20, 21]. However, our study is the first to focus on how time constraints and cannibalism are associated using a latitudinal gradient [42]. These cannibalism patterns may affect population dynamics, and whether it has stabilizing or de-stabilizing effects on population dynamics of the cannibalistic population depends on the age and size structure and whether density-dependent effects are due to causes other than cannibalism [43]. The studies reviewed in Claessen et al. [43] focused on biotic variables, however. In contrast, time constraint is an abiotic variable that does not necessarily have the same population dynamic effects as biotic variables since these tend to act on the population independently of population density. Exploring the combined effects of biotic and abiotic effects such as time constraints on population dynamics would be interesting.

## Additional file

Additional file 1: Contains Table A1 and Figs. A1–A3. (DOCX 2346 kb)
