# Supplementary material for: Cannibalism and activity rate in larval damselflies increase along a latitudinal gradient as a consequence of time constraints
Source: BMC Evol Biol. 2017 Jul 14;17:167. doi: 10.1186/s12862-017-1010-3 (PMC5513245; doi:10.1186/s12862-017-1010-3)
Supplement: Additional file 1: — Contains Table A1 and Figs. A1–A3. (DOCX 2346 kb) [file 12862_2017_1010_MOESM1_ESM.docx]

Additional file 1.

Cannibalism and activity rate in larval damselflies increase along a latitudinal gradient as a consequence of time constraints.

Authors: Szymon Sniegula, Maria J. Golab* Frank Johansson*

* Corresponding authors:

Frank Johansson

[frank.johansson@ebc.uu.se](mailto:frank.johansson@ebc.uu.se); Department of Ecology and Genetics, Uppsala University,
SE-751 05 Uppsala, Sweden

Maria J Golab

[marysiagolab@gmail.com](mailto:marysiagolab@gmail.com); Institute of Nature Conservation, Polish Academy of Sciences, 31-120 Krakow, Poland

Table A1 Results from the generalized linear model (GLM) analyses on number of larvae cannibalized by day 28 over the mean and total distance the larvae moved, and the number of days unitl one larva was left over the mean and total distance the larvae moved. Latitude and population within latitude was added as a factor and random effect, respectively in the generalized linear mixed models analyses (GLMM). The numerical results for interactions between latitude and activity traits are not shown since all were non-significant and exluded from the final models.

| Predictor | *DF* | *χ*^2^ | *P-value* |
| --- | --- | --- | --- |
| *No. of larvae cannibalized by day 28: GLM* |  |  |  |
| Mean distance moved | 1 | 14.842 | <0.001 |
| *No. of larvae cannibalized by day 28: GLM* |  |  |  |
| Total distance moved | 1 | 13.559 | <0.001 |
| *No. of larvae cannibalized by day 28: GLMM* |  |  |  |
| Mean distance moved | 1 | 1.639 | 0.201 |
| Latitude | 2 | 52.855 | <0.001 |
| *No. of larvae cannibalized by day 28: GLMM* |  |  |  |
| Total distance moved | 1 | 0.203 | 0.652 |
| Latitude | 2 | 50.686 | <0.001 |
| *No. of days until one larva left: GLM* |  |  |  |
| Mean distance moved | 1 | 24.054 | <0.001 |
| *No. of days until one larva left:GLM* |  |  |  |
| Total distance moved | 1 | 50.704 | <0.001 |
| *No. of days until one larva left: GLMM* |  |  |  |
| Latitude | 2 | 31.004 | <0.001 |
| Mean distance moved | 1 | 0.684 | 0.408 |
| *No. of days until one larva left:GLMM* |  |  |  |
| Latitude | 2 | 28.442 | <0.001 |
| Total distance moved | 1 | 0.138 | 0.710 |


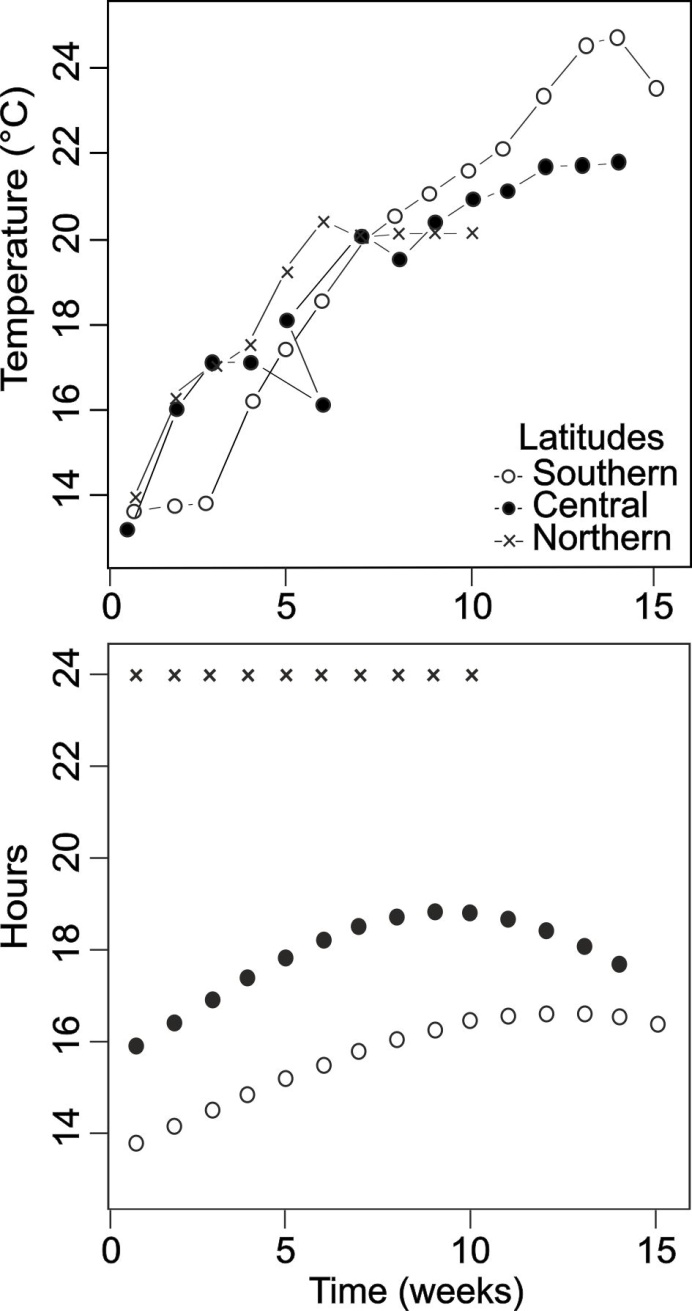


Figure A1 Mean weekly temperatures (top) and photoperiods (bottom) used during the experiment simulating natural conditions on northern, central and southern latitude populations of *Lestes* sponsa. Note that the latitude-specific curve reflects the duration of the cannibalism experiment until one larva was left.


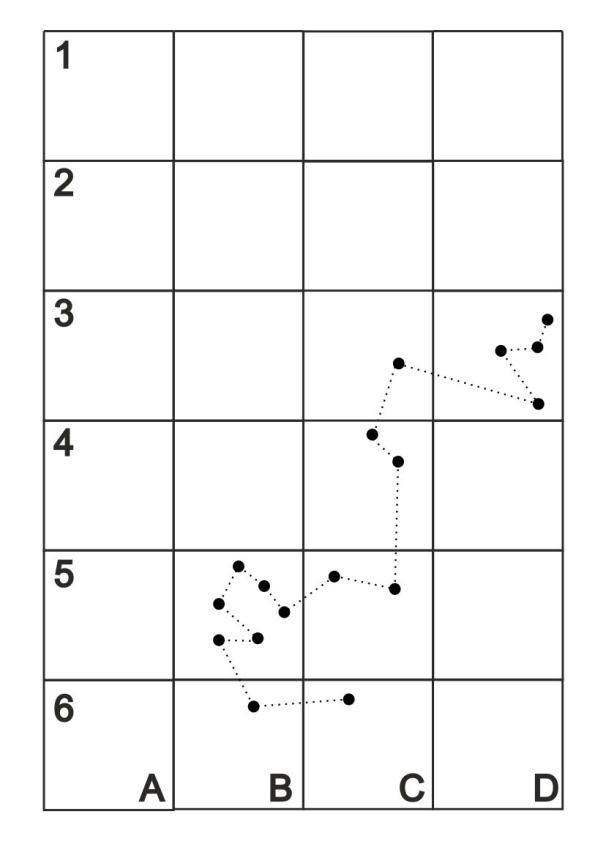


Figure A2 Larval moves marked on the grid used during the larval activity trials. The dots indicate larval head positions during a period of 20 min. In this example the larvae was scored 16 moves. The grid pattern was added as a references to aid determination of positions.


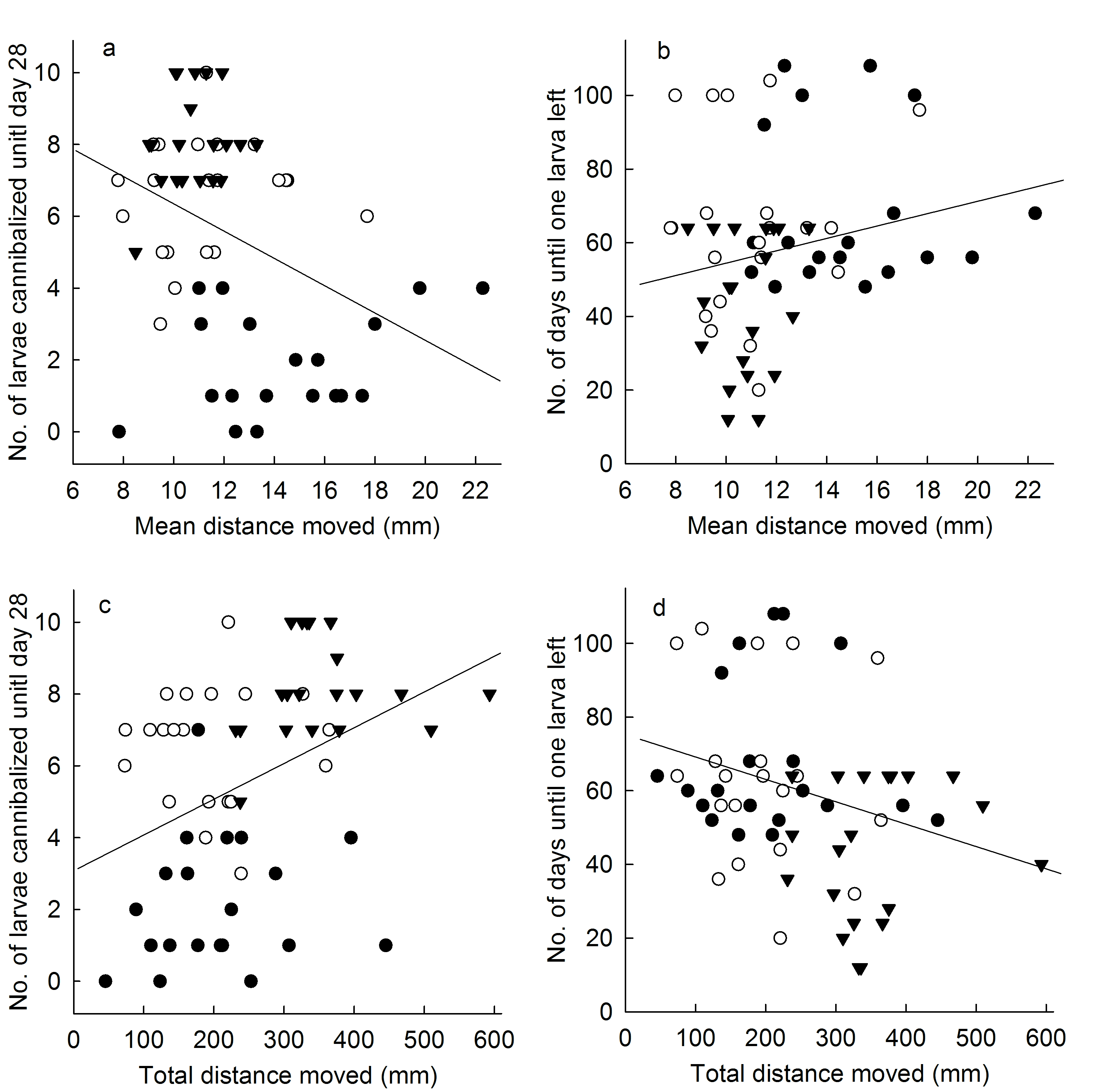


Figure A3 (a, b) Mean and (c, d) total distance moved regressed against (a, c) the number of larvae cannibalized by day 28 and (b, d) the number of days until one larva was left, respectively, of *Lestes sponsa* from (triangulars) northern, (open circles) central and (filled circles) southern latitudes during the experiment simulating natural conditions. Note that the regression line is based on the studied populations at all latitudes.
